# Admission Hypothermia and Factors Associated with Mortality among Admitted Hypothermic Preterm Neonates in Neonatal Intensive Care Units of Public Hospitals of Addis Ababa, Ethiopia

**DOI:** 10.1155/2022/8078628

**Published:** 2022-10-08

**Authors:** Fekadeselassie Belege Getaneh, Natnael Moges Misganaw, Dires Birhanu Mihretie, Zebenay Workneh Bitew

**Affiliations:** ^1^College of Medicine and Health Sciences, Wollo University, Dessie, Ethiopia; ^2^College of Health Sciences, Debre Tabor University, Debre Tabor, Ethiopia; ^3^College of Health Sciences, Dilla University, Dilla, Ethiopia; ^4^College of Health Sciences, St. Paul's Hospital Millennium Medical College, Addis Ababa, Ethiopia

## Abstract

**Background:**

Limited knowledge on the proportion of admission hypothermia and factors of death in hypothermic preterm neonates is hindering early and appropriate interventions in Ethiopia. Thus, studies on the proportion of admission hypothermia and factors of death in hypothermic preterm neonates are critical to enhancing preterm infants' survival.

**Methods:**

Hospital-based cross-sectional study was conducted on 398 participants using the systematic sampling method from October 10, 2021, to December 15, 2022. A pretested data extraction tool was used to collect data. EpiData version 4.6 and STATA version 16 were used for data entry and analysis. Multivariable logistic regression analysis evaluated the association between independent and outcome variables with a 95% confidence interval (CI). Hosmer and Lemeshow test and variance inflation factor were assessed to check model fitness and collinearity, respectively. *P*value < 0.05 was considered statistically significant.

**Result:**

Of the 398 admitted preterm neonates, 331(83.2%) had hypothermia at admission. Factors that were significantly associated with mortality included outborn babies [Adjusted hazard ratio (AOR) = 2.18 : 95% CI (1.03-4.62)], GA less than 32 weeks [AOR = 6.64 : 95% CI (1.87-13.58)], weight less than 1500 gram [AOR = 7.91 : 95% CI (1.21-15.38)], thrombocytopenia [AOR = 3.36 : 95% CI (1.49-7.58)], and kangaroo mother care [AOR = 0.38 : 95% CI (0.16-0.88)].

**Conclusion:**

The proportion of admission hypothermia was high. Outborn babies, birth weight less than 1500 gram, gestational age < 32 weeks, being thrombocytopenic, and lack of kangaroo mother care were identified as risk factors for hypothermic preterm neonatal mortality. Preterm labor prevention, improved inborn delivery, and kangaroo mother care may alleviate the high proportion of admission hypothermia and related mortalities in preterm neonates.

## 1. Introduction

Preterm babies are especially vulnerable and can become hypothermic within minutes, because of a high surface area-to-mass ratio, little subcutaneous adipose tissue, a thin stratum corneum, inadequate brown fat, and poor thermoregulation [1, 2]. Hypothermia is defined as skin (axillary) temperature less than 36.5°C [3]. World Health Organization (WHO) classifies 36.0°C to 36.4°C as cold stress or mild hypothermia, 32.0°C to 35.9°C as moderate hypothermia, and lower than 32.0°C as severe hypothermia, and the WHO advocates that neonatal body temperature should be maintained at 36.5°C to 37.5°C [4]. Hypothermia is one of the major risk factors for morbidity and mortality in the first 28 days of life and has been shown to be a risk factor for neonatal sepsis, intraventricular hemorrhage, and necrotizing enterocolitis [5]. According to recent research, every 1 degree Celsius drop in body temperature increases mortality by 80% [6].

Ninety percent of newborns in developing countries were hypothermic and 10.7% of neonates were severely hypothermic [5, 7]. Even though predisposing factors for hypothermia are easily preventable, the problem of hypothermia remains an unanswered question and it is highly prevalent in developing nations, including Sub-Saharan Africa [8–10]. In Ethiopia, hypothermia was the most common neonatal contributory cause; being present in (69.4%) of all neonatal deaths [11]. To the author's best knowledge, only a few studies assessed the relationship between admission hypothermia and neonatal morbidity and mortality [7, 8, 12–14]. Evidence-based analysis revealed that prevention and management of hypothermia could reduce 18%-42% of all-cause neonatal mortality or morbidity, globally [15].

To realize the UN sustainable development goal (SDG) 3 target 3.2.1 and the Ethiopian Health Sector Transformation Plan (HSTP) goal of decreasing neonatal mortality requires being aware of the burden, having evidence, and making the great effort to halt the effect of hypothermia [16, 17]. Therefore, this study will help to evaluate the essential care of baby in labor ward and NICUs and it will provide evidence to governmental and nongovernmental program developers and clinical researchers that may work on revising management guidelines for hypothermia so that neonatal mortality will significantly decrease.

## 2. Material and Methods

An institution-based cross-sectional study was carried out over 36 months, from September 1, 2018, to August 31, 2021, in five public NICUs in Addis Ababa, Ethiopia. There are twelve government hospitals in the town. Five belongs to the Addis Ababa Health Authority; four belongs to the Federal Ministry of Health, one to the Ministry of Education (AAU), and two to the Ethiopian Defense Force. Eleven of them have a neonatal unit. The study was conducted at Tikur Anbessa Specialized Hospital (which is the largest governmental teaching hospital in Ethiopia, located in the capital Addis Ababa. There are a total of 2 neonatologists and 30 nurses working in the unit. The average annual admission rate was 3985), Yekatit 12 Hospital Medical College (another teaching hospital in Addis Ababa) (which has a total of 38 nurses, 1 neonatologist fellow, and 3 pediatricians working in the unit. Average annual admission rate was being 2685), St. Peter Specialized Hospital, Ras Desta Damtew Memorial Hospital, and Gandhi Memorial Hospital (these hospitals belong to Addis Ababa Health Bureau with an average annual admission rate of 1740, 1550, and 3,700, respectively), all of which were chosen through a lottery system.

### 2.1. Population

All preterm neonates who were admitted to the neonatal intensive care units of public hospitals in Addis Ababa were the source population, and preterm neonates admitted to the neonatal intensive care units of selected public hospitals in Addis Ababa in the last three years were a study population.

### 2.2. Inclusion and Exclusion Criteria

All neonates who were admitted in the last three years in neonatal intensive care units were included in the sampling. To reduce the effects of other causes on study outcomes, we excluded infants with confirmed congenital anomalies. Infants who had incomplete medical information (admission temperature and outcome), referred from health facilities included in this study were also excluded.

### 2.3. Sample Size Determination and Sampling Procedure

First, the sample size was calculated based on the single population proportion formula by taking into account the following statistical assumptions *P* = proportion of hypothermia (pooled prevalence) = 57.2% [18], *Z* *α*/2 = the corresponding *Z* score of 95% CI and margin of error (5%), and the final sample size was 376. The other sample size was computed by using the double population proportion formula using Epi Info™ 7 with the assumptions: 95% CI, 80% power ratio of unexposed to exposed 1, the outcome in exposed = 50% outcome in unexposed 16.39%, and odd's ratio of 7.2 [10], and the sample size was 48. However, to increase the precision, we chose the higher sample size. By adding 10% was made for the missing data, and then the final sample size was 411.

**Figure 1:**
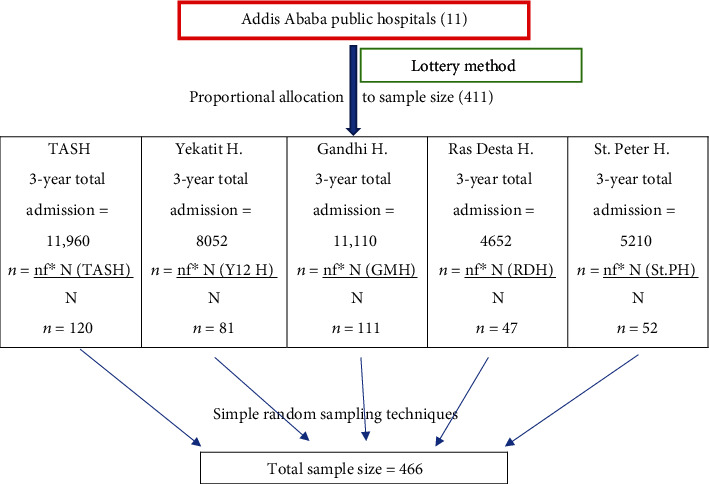
Schematic presentation of sampling procedure on proportion of admission hypothermia and outcomes of hypothermic preterm babies admitted in Addis Ababa public hospitals from 2018 to 2021, Addis Ababa, Ethiopia, 2022.

Based on the three-year total of the study period, the proportional allocation formula was used to pick study participants from each hospital and each year. The registration logbook was then used to identify the medical record numbers of preterm newborns. The study participants were then chosen using a computer-generated simple random sampling procedure from the isolated medical record numbers in each facility (See Figure 1).

### 2.4. Variables

#### 2.4.1. Dependent Variable

Admission hypothermia.

#### 2.4.2. Independent Variable

- *Neonatal conditions*: age at admission, birth weight, gestational age, crying at birth, and clinical problems at admission
- *Maternal condition*: parity, ANC follow-up, mode of delivery, and body contact
- *Health-related factors*: treatment provided like KMC, antibiotics, and respiratory support

### 2.5. Operational Definitions

- Admission normothermia: if admission temperature is ≥ 36.5and < 37.5 degree centigrade by axillary thermometer during the initial assessment of admission to NICU
- *Admission hypothermia*: if recorded temperature is < 36.5 degree centigrade by axillary thermometer during the initial assessment of admission to NICU
- *Inborn*: a newborn that was delivered from the study hospitals
- *Outborn*: a newborn that was delivered other than the study health facilities

### 2.6. Data Collection Tool and Procedure

Basic information, neonatal characteristics, newborn care-related factors, general medical condition, and the mother's delivery summary were all retrieved and collected from hospital records of preterm babies. The data extraction form was prepared based on the Federal Ministry of Health NICU management protocol [3], thermal protection WHO practical guide [4], and other peer-reviewed articles [8, 12, 19–21]. Five BSc (Bachelor of Science) neonatal nurses and two MSc (Masters of Science) holders were recruited for data collection and supervision, respectively. One-day training was given to data collectors and supervisors regarding the significance of the study, truthful completion of the checklist and ethical considerations to standardize the data collection. Finally, a chart review was performed from October 10, 2021, to December 15, 2022.

### 2.7. Data Quality Assurance

Data quality was assured by applying properly designed and pretested data collection tools. The data were collected by five BSc nurse experts. One-day training and clear orientation were provided on the process of data collection for data collectors. One week before the actual data collection, 5% of the study population took a pretest in another hospital to assess the clarity of the questions and the validity of the instrument. Two MSc professionals closely monitored and guided data collectors.

### 2.8. Data Processing and Analysis

After checking completeness, all questionnaires and checklists were coded and entered into EpiData version 4.6 before being exported to STATA version 16 for further analysis. Descriptive and summary statistics will be carried out and presented using texts, graphs, and tables. Bivariable and multivariable analyses were performed to determine the relationship between baseline and explanatory variables. Variables with a *P* value less than 0.1 in bivariable logistic regression were entered into multivariable logistic regression to adjust the effect of confounders on the outcome variable. The degree of association was determined using the odds ratio with 95% confidence interval. A *P* value of less than 0.05 was considered significant.

## 3. Results

### 3.1. Socio-Demographic and Obstetrics Characteristics of the Mothers

A total of 398 neonatal charts were included, with a 96.8% response rate. The mean age of the mothers was 27.5 years (± SD) 4.69. The majority of the age group was between 20 and 34 (88.44%) years of age. Three hundred forty-nine (87.69%) were urban residents. Regarding the obstetric history of the mother, only 9 (2.2%) had no antenatal visits during their pregnancy. More than half (59.05%) of mothers were multiparous, and two hundred eighty-two (70.85%) carried singleton pregnancies. In 210 (52.76%) of the cases, the modes of delivery were spontaneous vertex delivery, with 178 (44.72 percent) being cesarean delivery (C-section). Nearly half of the mothers, 188 (47.24%) and 196 (49.25%), gave birth at the same facility where their infant was admitted and at nighttime, respectively, while 11 (2.76%) of mothers gave birth at home. Among all admitted neonates, 117 (29.4%) were born from pregnancy-induced hypertensive pregnant women and 33 (8.29%) from moms who had an antepartum hemorrhage. Seven mothers (1.76%) developed oligohydramnios, and 11 (2.76%) showed signs of chorioamnionitis (See.  Table 1).

### 3.2. Characteristics of the Preterm Neonates Admitted to the NICU

Two hundred sixteen (54.27%) and 382 (95.98%) newborns were male and admitted within 24 hours of postnatal age, respectively. Out of 16 neonates admitted after 24 hours of age, 13(81.25%) had hypothermia at admission. The mean (+ SD) age of the neonate at presentation was 4.2(17.7) hours. Only 33 (8.29%) of preterm neonates had a normal birth weight (2500-3999 g). Nearly half (49.75%) of the babies born between 32 weeks and 36 weeks of gestational age and the majority (88%) of the baby were appropriate for their gestational age. Sixty-one present (245) newborns cried immediately after birth.

### 3.3. Clinical and Treatment Characteristics of Hypothermic Preterm Neonates

More than half, (56.1% and 56.7%) of the dead hypothermic preterm babies were male and very-low-birth-weight babies, respectively. Among the comorbidities identified at presentation, two hundred fifty-two (76.1%) newborns suffered from respiratory distress, and 69 (20.9%) had a sign of perinatal asphyxia (based on the 5 min Apgar score). Suspected sepsis, hyperbilirubinemia, thrombocytopenia, suspected necrotizing enterocolitis, and recurrent hypoglycemia were additional medical diagnoses of hypothermic preterm babies, with 83.9%, 53.5%, 36.9%, 25.4%, and 7.3%, respectively. The majority of the babies (93.9%) had received antibiotics and two hundred eleven (63.7%) and 103 (31.2%) of preterm babies had continuous positive airway pressure and direct oxygen respiratory support, respectively. Out of 81 (24.5%) of hypothermic preterm babies who got kangaroo mother care, twenty-nine percent of them recovered and were discharged alive from NICUs (See Table 2).

### 3.4. Proportion of Admission Hypothermia and Outcomes of Hypothermic Preterm Babies

**Figure 2:**
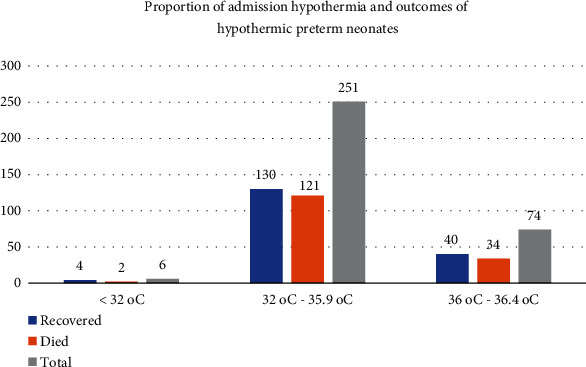
Proportion of admission hypothermia and outcomes of hypothermic preterm babies admitted in Addis Ababa public hospitals from 2018 to 2021, Addis Ababa, Ethiopia, 2022.

A total of 331 (83.17%) admitted preterm neonates had admission hypothermia (temperatures lower than 36.5°C), with 74 (18.59%) having mild hypothermia, 251 (63.07%) having moderate hypothermia, and 6 (1.51%) having severe hypothermia group. The average (+ SD) admission temperature was 35.2°C (1.3°C), with temperatures ranging from 32°C to 40°C. Regarding the outcomes of hypothermic preterm babies, 157 (47.43%) died and 174 (52.56%) were improved and discharged from NICUs (See Figure 2).

### 3.5. Factors Associated with Mortality of Hypothermic Preterm Neonates

After checking the model for multicollinearity and model fitness; variables with a *P*value < 0.1 in the bivariate analysis were considered in the final model. Hence, the multivariable logistic regression analysis showed that babies born outside the admitting institution, birth weight less than 1500 gram, gestational age < 32 weeks, being thrombocytopenic, and lack of kangaroo mother care in NICUs were the independent predictors of mortality.

Preterm neonates born from outside the admitting health institution had 2.2 times greater odds of neonatal mortality compared to inborn babies [AOR = 2.18 : 95% CI (1.03-4.62)]. The odds of dying were 6.6 and 7.9 times higher in GA less than 32 weeks and very low birth weight hypothermic preterm neonates, respectively [AOR = 6.64 : 95% CI (1.87-13.58)] and [AOR = 7.91 : 95% CI (1.21-15.38)]. Thrombocytopenic preterm neonates had 3.4 times greater odds of mortality compared to nonthrombocytopenic neonates [AOR = 3.36 : 95% CI (1.49-7.58)]. Moreover, neonates who got kangaroo mother care were 62% less likely to die compared to neonates who did not [AOR = 0.38 : 95% CI (0.16-0.88)] (See.  Table 3).

## 4. Discussion

This study shows the proportion of hypothermia at admission and factors associated with hypothermic preterm neonatal deaths for infants admitted to public hospitals in Addis Ababa, Ethiopia. We show that the proportion of admission hypothermia was 83.17%. This finding was consistent with a multicountry study (China (88.2%) [12], Malawi (77%) [8], and Korea (74.3%) [14], but higher than the study conducted in Ghana [10] and different parts of Ethiopia [18, 20, 22, 23]. Moreover, the obvious reasons of sample size, study participants and study period differences, temperature measurement time, differences in hospital set-ups (equipment available and skilled personnel), and socioeconomic differences across regions might be the possible explanation for the discrepancy.

According to this study's finding, hypothermic very preterm and VLBW babies had a greater chance of mortality compared to term and normal birth weight preterm infants. This finding has also been reported in other settings [7, 8, 12]. This is because babies with small gestational age or birth weight have a large surface area per unit of body weight, immature hypothalamic thermal control, immature and thin skin, and low subcutaneous and brown fat. Due to this, majority of them had hypothermia [2]. Hypothermia also leads to increased oxygen consumption, which leads to hypoxemia, pulmonary vasoconstriction, the reduced release of pulmonary surfactant, and eventual death [5, 6].

Thrombocytopenia was another independent factor determined in this study that had a significant effect on the death of hypothermic preterm neonates. This statistically significant association between thrombocytopenia and hospital mortality reaffirms the importance of early diagnosis and management of thrombocytopenia in determining neonatal outcomes. Thrombocytopenia is mostly common in hypothermic preterm babies because of the increased risk of sepsis, necrotizing enterocolitis, and slowing down of coagulation enzymes, disordered fibrinolysis, and disruption of platelet function [4, 19, 24]. Moreover, in our setting, management of thrombocytopenic preterm infants is still deficient (gap in accessibility, appropriate storage, and proper administration of blood components), contributing to poor outcomes.

This study also revealed outborn preterm neonates had 2.2 times more likely to die than neonates born in the same institution of admission health facilities (inborn babies). Proper hypothermia prevention on delivery, appropriate baby wrapping, well-heated transportation to the NICU (keeping skin contact with their mothers' skin during transportation and use of plastic bag wrap for small babies), these were low-cost interventions that can impact clinical outcome [25]. All of these are inadequately implemented in our study setting. Furthermore, neonates who got kangaroo mother care had 62% less chance of dying than those who did not get the care. Even though our study had not considered the type of KMC provided, the finding is analogous to different studies [8, 9, 26, 27]. Kangaroo care can help to stabilize heart rate, improve oxygen saturation and respiratory rate, improve infant lactation [28], and have positive effects on neurological, cognitive, emotional, behavioral, and social development in the short and long term [29]. In addition, KMC improves heat generation via oxidative phosphorylation, enables slow rewarming (this decreases oxygen consumption and apnea), and obtains enough calories from its mother's milk [30].

## 5. Limitation of the Study

Since it was a chart review, the study did not address all necessary variables like the temperature of labor wards and NICUs, or other treatment or service-related predictors. And also, the measurement of temperature was only based on a single measurement record, and the instrument used, the person who took the measurement, the site, and time of measurement taken might not be similar for all neonates, which may undifferentiable bias the result. Finally, the calibration of thermometers (the measurement had not been cross-checked with reference thermometers).

## 6. Conclusions

The proportion of admission hypothermia in the study area was relatively high. Outborn babies, birth weight less than 1500gram, gestational age < 32 weeks, being thrombocytopenic, and lack of kangaroo mother care were identified as factors that significantly increase the mortality of hypothermic preterm neonates at admission. Preterm labor prevention, improve inborn delivery, and kangaroo mother care may alleviate the high proportion of admission hypothermia and related mortalities in preterm neonates.

## Figures and Tables

**Table 1:** Cross-tabulation showing the preterm neonate and obstetrics characteristics with outcomes of hypothermic preterm babies in Addis Ababa public hospitals, 2022.

Variables	Categories	Outcomes of hypothermic preterm babies (*n* = 311)		Total, *N* (%)	*X* ^2^ test	*P* value
		Discharged, *N* (%)	Died, *N* (%)			
Sex of the baby	Male	89 (51.1)	88 (56.1)	177 (53.5)	0.79	0.372
	Female	85 (48.9)	69 (43.9)	154 (46.5)		

Birth weight in grams	<1500	18 (10.3)	89 (56.7)	107 (32.3)	84.46	<0.01
	1500-2499	135 (77.6)	65 (41.4)	200 (60.4)		
	≥2500	21 (12.1)	3 (1.9)	24 (7.3)		

Gestational age in weeks	<32	10 (5.8)	87 (55.4)	97 (29.3)	104.51	<0.01
	32–35 + 7	104 (59.8)	57 (36.3)	161 (48.6)		
	≥ 36	60 (34.4)	13 (8.3)	73 (22.1)		

Age at the time of presentation in hours	< 24	169 (97.1)	149 (94.9)	318 (96.1)	*Nf*	*Nf*
	24-72	5 (2.9)	6 (3.8)	11 (3.3)		
	>72	0 (0.0)	2 (1.3)	2 (0.6)		

Weight for age	SGA	14 (8.05)	24 (15.29)	38 (11.48)	*Nf*	*Nf*
	AGA	158 (90.8)	132 (84.08)	290 (87.61)		
	LGA	2 (1.15)	1 (0.64)	3 (0.91)		

Place of delivery	Inborn	79 (45.4)	78 (49.7)	157 (47.4)	*Nf*	*Nf*
	Outborn	94 (54)	72 (45.8)	166 (50.1)		
	Home	1 (0.6)	7 (4.5)	8 (2.5)		

Mode of delivery	SVD	87 (50)	86 (54.8)	173 (52.3)	*Nf*	*Nf*
	C/S	82 (47.1)	68 (43.3)	150 (45.3)		
	AVD	5 (2.9)	3 (1.9)	8 (2.4)		

Time of delivery	Daytime	84 (48.3)	86 (54.8)	170 (51.4)	1.39	0.237
	Nighttime	90 (51.7)	71 (45.2)	161 (48.64)		

Crying immediately after birth	Yes	112 (64.4)	86 (54.8)	198 (59.8)	3.15	0.076
	No	62 (35.6)	71 (45.2)	133 (40.2)		

*xNf*: not fulfill chi-square assumption; NB: -; SGA: small for gestational age; AGA: appropriate for gestational age; LGA: large for gestational age; SVD: spontaneous vertex delivery; C/S: cesarean-section; AVD: assisted vaginal delivery.

**Table 2:** Cross-tabulation showing the clinical and treatment characteristics of babies with outcomes of hypothermic preterm babies in Addis Ababa public hospitals, 2022.

Variables	Categories	Outcomes of hypothermic preterm babies (*n* = 311)		Total, *N* (%)	*X* ^2^ test	*P* value
		Discharged, *N* (%)	Died, *N* (%)			
Respiratory distress	Yes	109 (62.6)	143 (91.1)	252 (76.1)	36.73	<0.01
	No	65 (37.4)	14 (8.9)	79 (23.9)		
Suspected sepsis	Yes	133 (76.4)	145 (92.4)	278 (83.9)	15.55	<0.01
	No	41 (23.6)	12 (7.6)	53 (16.1)		
Perinatal asphyxia	Yes	15 (8.6)	54 (34.4)	69 (20.9)	33.23	<0.01
	No	159 (91.4)	103 (65.6)	262 (79.1)		
Necrotizing enterocolitis	Yes	9 (5.2)	75 (47.8)	84 (25.4)	79.08	<0.01
	No	165 (94.8)	82 (52.2)	247 (74.6)		
Proven sepsis	Yes	20 (11.5)	63 (40.1)	83 (25.1)	36.01	<0.01
	No	154 (88.5)	94 (59.9)	248 (74.9)		
Hyperbilirubinemia	Yes	80 (45.9)	97 (61.8)	177 (53.5)	8.29	0.004
	No	94 (54.1)	60 (38.2)	154 (46.5)		
Recurrent hypoglycemia	Yes	13 (7.5)	11 (7.1)	24 (7.3)	0.02	0.871
	No	161 (92.5)	146 (92.9)	307 (92.7)		
Thrombocytopenia	Yes	23 (13.2)	99 (63.1)	122 (36.9)	88.08	<0.01
	No	151 (86.8)	58 (36.9)	209 (63.1)		
Kangaroo mother care	No	123 (70.7)	127 (80.9)	250 (75.5)	4.64	0.031
	Yes	51 (29.3)	30 (19.1)	81 (24.5)		
Respiratory support	CPAP	68 (39.1)	143 (91.1)	211 (63.7)	96.83	<0.01
	Oxygen	90 (51.7)	13 (8.3)	103 (31.2)		
	No any	16 (9.2)	1 (0.6)	17 (5.14)		
Antibiotics	Yes	156 (89.7)	155 (98.7)	311 (93.9)	11.96	<0.01
	No	18 (10.3)	2 (1.3)	20 (6.1)		

NB: -; CPAP: continuous positive airway pressure.

**Table 3:** Bivariable and multivariable logistics regression analysis results of hypothermic preterm babies who were admitted at neonatal intensive care units of Addis Ababa public hospitals, Ethiopia, 2022 [*n* = 311].

Covariates		COR (95% CI)	*P* value	AOR (95% CI)	*P* value
Parity	Multiparity	1		1	
	Primiparity	1.49 (0.96-2.31)	0.075	1.22 (0.57-2.59)	0.594

Place of delivery	Inborn	1		1	
	Outborn	0.82 (0.53-1.28)	0.039	2.18 (1.03-4.62)	0.041^∗^
	Home	2.02 (0.17-22.79)	0.568	39.9 (0.79-200.3)	0.065

Pregnancy-induced hypertension	No	1		1	
	Yes	3.13 (1.91-5.15)	0.001	2.16 (0.97-4.80)	0.058

Birth weight	≥2500 gram	1		1	
	1500–2499 gm	3.37 (0.97-11.71)	0.056	1.74 (0.32-9.41)	0.518
	<1500 gram	34.6 (9.32-58.46)	0.001	7.91 (1.21-15.4)	0.030^∗^

Gestational age	≥ 36 weeks	1		1	
	32 wks–35 wks	2.52 (1.28-4.99)	0.008	1.42 (0.52-4.05)	0.466
	<32 weeks	40.15 (16.52-97.5)	0.001	6.64 (1.87-13.6)	0.003^∗∗^

Weight for age	AGA	1		1	
	LGA	0.59 (0.05-6.67)	0.677	0.61 (0.01-20.2)	0.867
	SGA	2.05 (1.02-4.12)	0.044	1.42 (0.44-4.58)	0.551

Crying immediately after birth	Yes	1		1	
	No	1.49 (0.95-2.31)	0.076	1.10 (0.46-3.13)	0.795

Respiratory distress	No	1		1	
	Yes	6.09 (3.24-11.42)	0.001	1.20 (0.46-3.13)	0.708

Suspected sepsis	No	1		1	
	Yes	3.72 (1.87-7.38)	0.001	2.29 (0.76-6.88)	0.138

Perinatal asphyxia	No	1		1	
	Yes	5.24 (2.80-9.81)	0.001	2.40 (0.87-6.55)	0.087

Necrotizing enterocolitis	No	1		1	
	Yes	9.67 (4.85-19.26)	0.001	2.39 (0.89-6.44)	0.083

Proven sepsis	No	1		1	
	Yes	5.16 (2.93-9.07)	0.001	1.68 (0.65-4.36)	0.286

Hyperbilirubinemia	No	1		1	
	Yes	1.89 (1.22-2.94)	0.004	0.74 (0.35-1.58)	0.445

Thrombocytopenia	No	1		1	
	Yes	11.2 (6.49-19.33)	0.001	**3.36 (1.49-7.5**8**)**	0.003^∗∗^

Antibiotics	No	1		1	
	Yes	8.94 (2.04-19.19)	0.004	2.93 (0.22-17.8)	0.409

Kangaroo mother care	No	1		1	
	Yes	0.56 (0.34-0.95)	0.032	**0**.38 **(0**.16-0.88**)**	0.024^∗^

Respiratory support	No any	1		1	
	CPAP	33.64 (4.37-58.97)	0.001	4.66 (0.44-9.10)	0.199
	Oxygen	2.31 (0.28-18.91)	0.435	1.45 (0.14-14.9)	0.751

NB*: -*^∗^significant (*P*value < 0.05), ^∗∗^significant (*P*value < 0.01), and OR = 1 is reference variable. NB: -; SGA: small for gestational age; AGA: appropriate for gestational age; LGA: large for gestational age; CPAP: continuous positive airway pressure.

## Data Availability

The data sets used and/or analyzed during the current study are available from the corresponding author on reasonable request.

## Abbreviations

AAU: Addis Ababa University
AGA: Appropriate for gestational age
AOR: Adjusted odd's ratio
ANC: Antenatal care
APGAR: Appearance, pulse, grimace, activity, respiration
CPAP: Continuous positive airway pressure
GA: Gestational age
AVD: Assisted vaginal delivery
C/s: Cesarean-section
HMIS: Health management information system
KMC: Kangaroo mother care
LGA: Large for gestational age
NEC: Necrotizing enterocolitis
NICU: Neonatal intensive care unit
SGA: Small for gestational age
SVD: Spontaneous vertex delivery.
